# Analgesic Efficacy of Gabapentin in Patients Undergoing Carpal Tunnel Release Surgery: A Systematic Review and Meta-Analysis of Randomized Placebo-Controlled Trials

**DOI:** 10.7759/cureus.77808

**Published:** 2025-01-22

**Authors:** Muteb N Alotaibi, Ohood Y Alasmari, Omar E Elshaer, Ibrahim S Allehaimeed, Hajar A Alharbi, Amal A Alsubaiei, Abdullah M Alharran, Abdulmuhsen Alqallaf, Mohammed Alshammari, Abdullah Alhuwailah, Ahmed F AlFaleh

**Affiliations:** 1 Medicine and Surgery, Alfaisal University, Riyadh, SAU; 2 Medicine and Surgery, Princess Nourah bint Abdulrahman University, Riyadh, SAU; 3 General Medicine, Qassim Health Cluster, Buraydah, SAU; 4 Medicine and Surgery, Qassim University, Buraydah, SAU; 5 Medicine and Surgery, Arabian Gulf University, Manama, BHR; 6 Medicine and Surgery, Kuwait Institute for Medical Specializations, Kuwait City, KWT; 7 Medicine and Surgery, Faculty of Medicine, Alexandria University, Alexandria, EGY; 8 Orthopedic Surgery, King Abdullah bin Abdulaziz University Hospital, Riyadh, SAU

**Keywords:** carpal tunnel release, carpal tunnel syndrome, gabapentin, meta-analysis, pain

## Abstract

Carpal tunnel syndrome (CTS) results from median nerve compression and may lead to significant pain. Surgical management through release is the gold standard approach for severe CTS patients. Gabapentin is used as an analgesic drug, but data on its postoperative effects on pain assessment and safety measures are unclear. We aimed to assess the clinical effectiveness of gabapentin in patients undergoing CTS release surgery. We searched PubMed, Scopus, Web of Science (WOS), and the Cochrane Library for randomized controlled trials (RCTs) addressing the effectiveness of gabapentin in patients with CTS release until September 2024. The primary outcome was the assessment of postoperative pain at one, six, 12, and 24 hours by a visual analog scale (VAS). Other specific outcomes were adverse events. Data were pooled as effect sizes (mean difference (MD) or odds ratio (OR)) with their 95% confidence interval (CI) in a random-effects model using Stata/MP 18. Three RCTs comprising 205 patients were included in the pooled meta-analysis. Gabapentin significantly reduced postoperative pain at six, 12, and 24 hours compared to placebo (MD = -0.6, 95% CI: -0.63 to 0.57, p < 0.001; MD = -2.14, 95% CI: -2.18 to -2.1, p < 0.001; and MD = -1.41, 95% CI: -1.82 to -0.99, p < 0.001, respectively). On the other hand, no significant differences were observed regarding other studied outcomes (i.e., safety) between the two groups. This pooled meta-analysis of 205 patients revealed that gabapentin was associated with reduced pain postoperatively at 6, 12, and 24 hours with comparable rates of adverse events compared to placebo. Further RCTs are warranted to validate the current findings.

## Introduction and background

Carpal tunnel syndrome (CTS) is a prevalent clinical condition caused by compression of the median nerve as it traverses the carpal tunnel in the wrist [1]. This compression renders the nerve particularly susceptible to injury and is clinically associated with symptoms such as hand numbness, tingling, nocturnal pain, and significant weakness in the hand and fingers, which may ultimately result in functional impairment [1].

The management of CTS encompasses both surgical and non-surgical approaches. Non-surgical interventions include wrist splinting, local steroid injections, pharmacological therapies for symptom relief, and activity modification [2]. However, for severe cases, surgical intervention remains the gold standard [2]. Despite its efficacy, the surgical approach is associated with potential complications, including postoperative pain, infections, tendon injuries, and nerve damage.

Gabapentin, an anticonvulsant and analgesic medication, has demonstrated promising results in managing neuralgia and other neurological disorders [3-5]. It has garnered significant attention in pain management due to its therapeutic modulation of calcium channels, leading to the inhibition of neurotransmitter release, reduced neuronal excitability, and attenuation of pain perception [3-5]. Although previous studies have reported favorable outcomes with gabapentin in patients with CTS [6-8], its specific role in managing postoperative pain following carpal tunnel release surgery remains unclear.

Therefore, this systematic review and meta-analysis aim to evaluate the analgesic efficacy of gabapentin in CTS patients undergoing release surgery, providing a comprehensive clinical assessment of its potential benefits.

## Review

Methods and materials

We performed this systematic review and meta-analysis in accordance with the Preferred Reporting Items for Systematic Reviews and Meta-Analyses (PRISMA) statement for meta-analyses [9] and guidelines reported in the Cochrane Handbook for Systematic Reviews and Meta-Analyses [10].

Search Strategy and Data Sources

Four databases, PubMed, Scopus, Web of Science (WOS), and Cochrane Library, were retrieved until December 2024 for randomized controlled trials (RCTs) using the following search strategy: ((“carpal tunnel syndrome release” OR “median nerve compression release”) AND (“gabapentin” OR “Neurontin” OR “Convalis” OR “Gralise”)).

Eligibility Criteria and Study Selection

All RCTs assessing the effectiveness of gabapentin as an analgesic drug were included if they meet our PICO criteria as follows: I) population: patients with CTS undergoing surgical release, II) intervention: gabapentin regardless of the dose, III) comparator: placebo as the control group, and IV) outcomes: postoperative pain as a primary outcome of interest. Then, the incidence of adverse events such as dizziness, drowsiness, tinnitus, and metallic taste were studied as the secondary outcomes.

We excluded single-arm studies, narrative reviews, conference abstracts, unpublished data, or studies that did not assess our outcomes of interest. Moreover, due to the nature of this type of surgery where intra-operative analgesia is not needed as intravenous regional anesthesia (IVRA) is sufficient for local anesthesia, studies assessed gabapentin in combination with other drugs such as celecoxib or acetaminophen were excluded. 

Outcomes

The primary outcome of interest was the assessment of postoperative pain at one, six, 12, and 24 hours from surgery using a 10-point scale like the visual analog scale (VAS) for pain measurements. The VAS tool was used globally for acute and chronic pain conditions [11]. The scale is a 10-point score, with “10” indicating the worst pain ever and “0” indicating no pain at all. Other studied outcomes were adverse events such as dizziness, drowsiness, tinnitus, and metallic taste.

Quality Assessment and Data Extraction

The Cochrane risk-of-bias assessment tool 2 (ROB-2) was used to evaluate the ROB in the included RCTs [12]. The tool includes five domains: randomization, deviations, missing data, outcome measurement, and result selection. Two reviewers independently assessed the ROB tool and labeled it as "low risk," "high risk," or "some concerns," with discrepancies resolved by a third author through consensus. A standardized Excel sheet was used in data extraction, which consisted of baseline characteristics of the included studies and patients, and the studied outcomes.

Statistical Analysis

Continuous data were pooled as mean differences (MDs) with their 95% confidence intervals (CI) in a random-effect model using DerSimonian-Liard methodology, and pooled odds ratios (ORs) and 95% CI were used as the pooled effect estimates of the dichotomous data. The statistical heterogeneity was assessed by I-squared (I²) statistics, and p-value of <0.05 and I-square values ≥50% were used as the cut-off point of significance. The package “meta esize” was used on Stata Statistical Software release 18 (StataCorp LLC, College Station, TX) to pool the effect estimates and their corresponding 95% CI.

Results

Search Results

**Figure 1:**
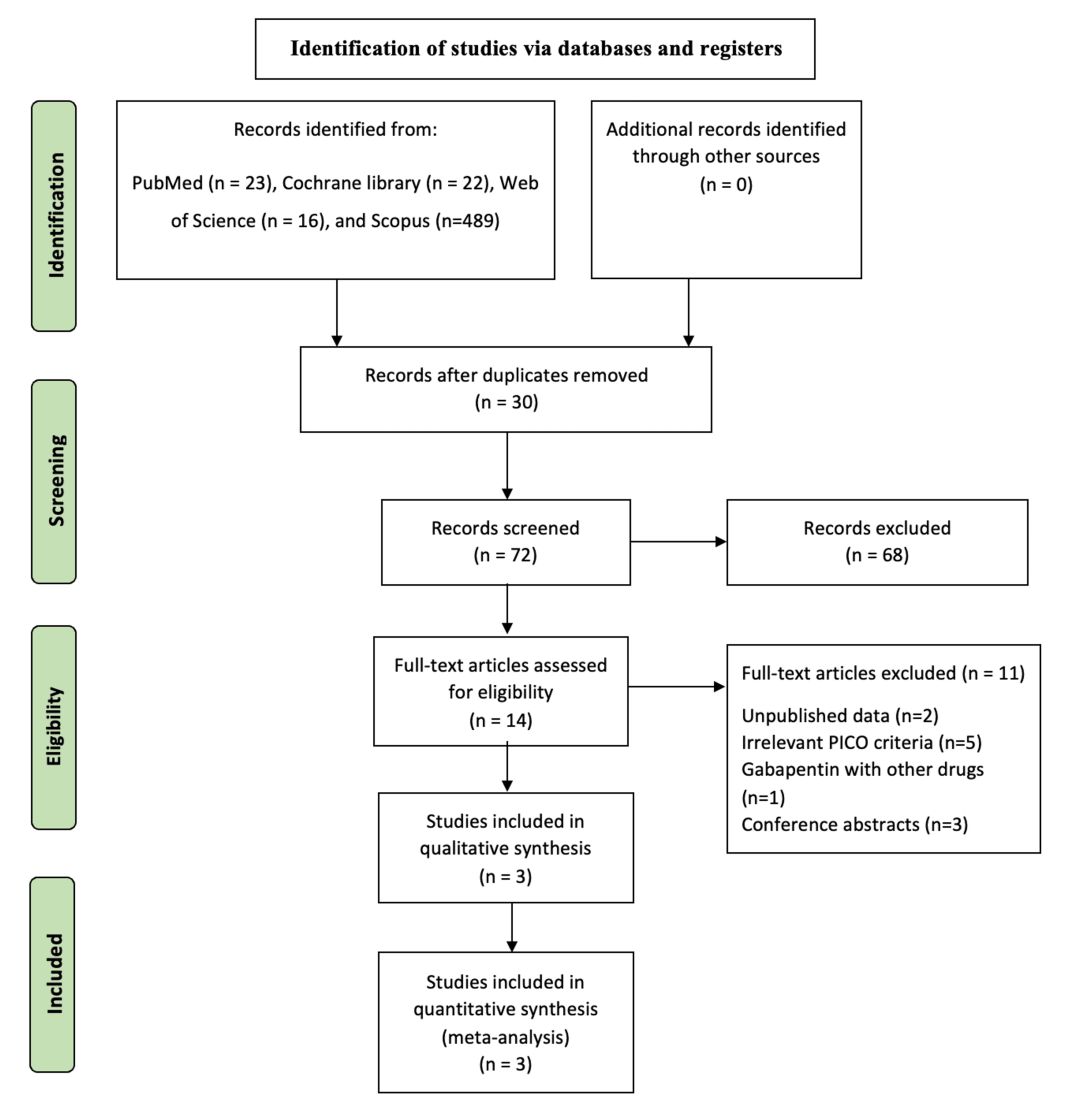
Preferred Reporting Items for Systematic Reviews and Meta-Analyses (PRISMA) flow diagram of the selection process.

A total of 102 references were retrieved from databases. After removing 30 references as duplicates, 72 studies were considered for screening. A total of 14 studies were eligible for the full-text screening phase after title and abstract screening. Eleven studies were excluded, and finally, three studies that matched our PICOs were included in the final analysis [13-15]. The PRISMA flow diagram for study selection is shown in Figure 1.

Study Characteristics

Three RCTs comprising 205 patients were included in the final analysis. Of these, 123 patients were assigned to gabapentin and 82 patients were assigned to placebo. Two studies [13,14] used a 600 mg dose of gabapentin, while the other study [15] used 100 mg and 300 mg as the standard doses. The mean duration of CTS release surgery procedure and anesthesia were comparable between the two studied groups. Detailed summary and baseline characteristics are shown in Tables 1-2.

**Table 1:** Summary data of the included studies. CTS: carpal tunnel syndrome

Study ID	Study design	Country	Sample size	Type of surgery	Trial arm		Type of anesthesia	Gabapentin dose	Follow-up
					Intervention	Control			
Eidi, 2023 [15]	Randomized placebo-controlled trial	Iran	n = 120	CTS release surgery	Gabapentin	Placebo	Not reported	100 mg and 300 mg	24 hours
Georgeto, 2021 [14]	Randomized placebo-controlled trial	Brazil	n = 45	CTS release surgery	Gabapentin	Placebo	Local anesthesia	600 mg	14 days
Sadatsune, 2016 [13]	Randomized placebo-controlled trial	Brazil	n = 40	CTS release surgery	Gabapentin	Placebo	Intravenous regional anesthesia	600 mg	6 months

**Table 2:** Baseline characteristics of the included patients.

Study ID	Group	Sample size	Age (year)	Male/female	Timing of anesthesia	Duration of the surgery, minutes	
							Eidi, 2023 [15]	Gabapentin 100 mg	n = 40	(18-65)	Not reported	1 hour pre-operatively	Not reported	
Gabapentin 300 mg	n = 40	(18-65)	Not reported	Not reported								
Placebo	n = 40	(18-65)	Not reported	Not reported								
Georgeto, 2021 [14]	Gabapentin 600 mg	n = 23	51.09 ±8.67	[1/22]	1 hour post-operatively	54.33 ± 10.58	
	Placebo	n = 22	52.41 ± 12.01	[2/20]			
Sadatsune, 2016 [13]	Gabapentin 600 mg	n = 20	51.5 (48.5-54)	[0/20]	1 hour post-operatively	33.06 ± 10.86	
	Placebo	n = 20	52.1 (46-56.7)	[0/20]		33.06 ± 16.90	

**Figure 2:**
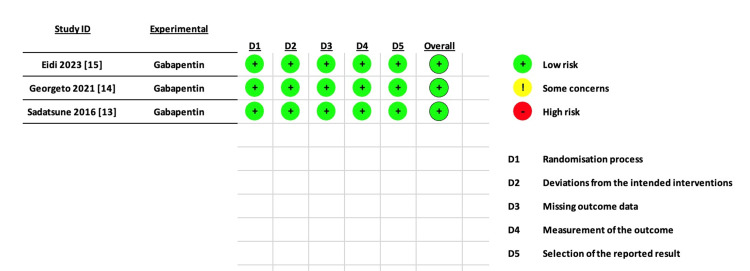
Detailed summary of risk of bias 2 (ROB-2)

All studies were at low risk of bias regarding the ROB-2 tool assessment. Figure 2 shows a detailed summary of ROB-2.

Outcomes

**Figure 3:**
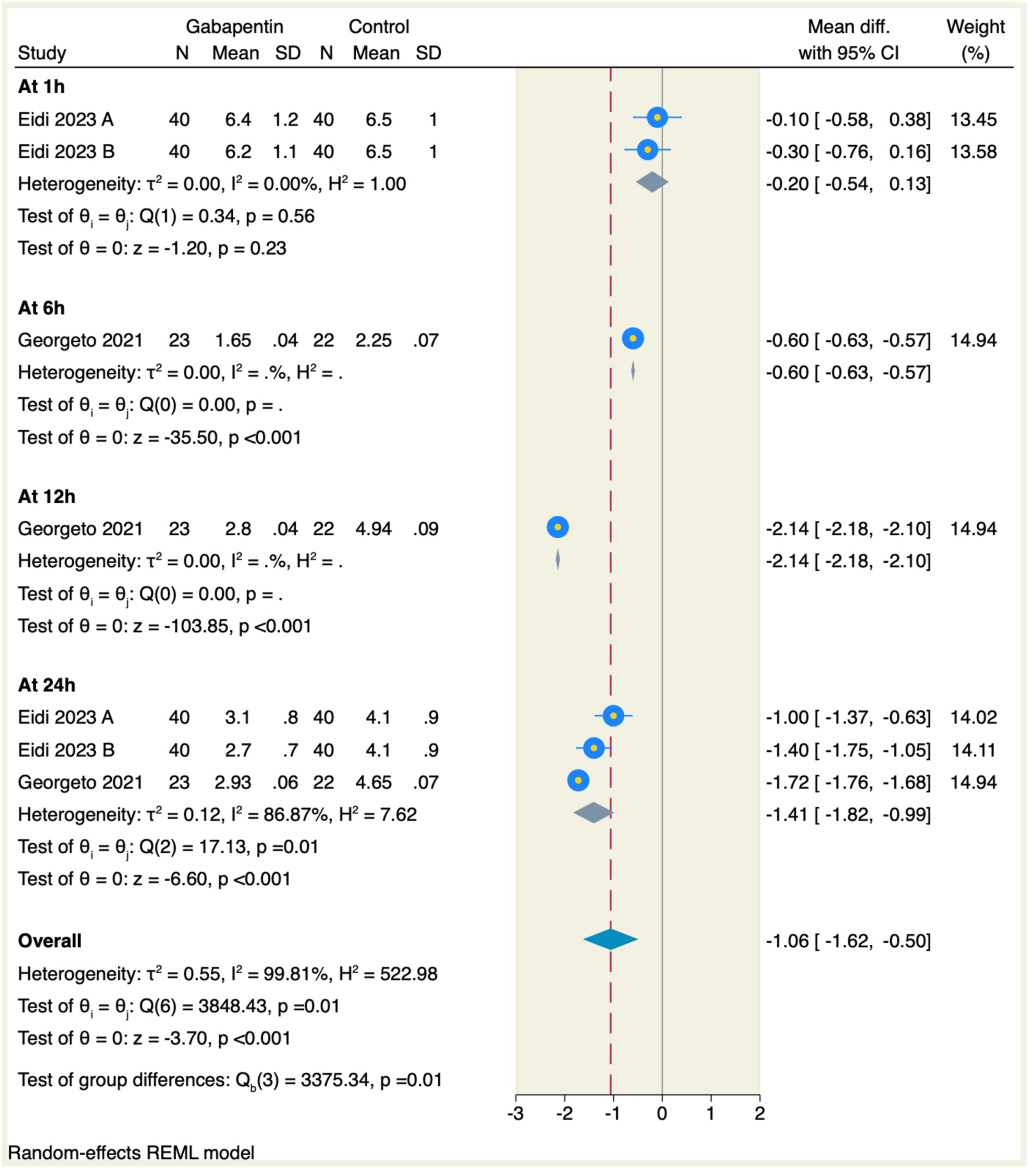
Forest plot of postoperative pain assessment at different time points (visual analog scale (VAS))

All of the included studies assessed the postoperative pain of which gabapentin significantly reduced the postoperative pain at six, 12, and 24 hours compared to the placebo group, with the following values, respectively (MD = -0.6, 95% CI: -0.63 to -0.57, p < 0.001; -2.14, 95% CI: -2.18 to -2.1, p < 0.001; and -1.41, 95% CI: -1.82 to -0.99, p < 0.001; I2 = 86.87, p = 0.01). On the other hand, there was no significant difference between gabapentin and placebo regarding the postoperative pain at one hour (MD = -0.2, 95% CI: -0.54 to 0.13, p = 0.23; I2 = 0.00, p = 0.56), as shown in Figure 3.

**Figure 4:**
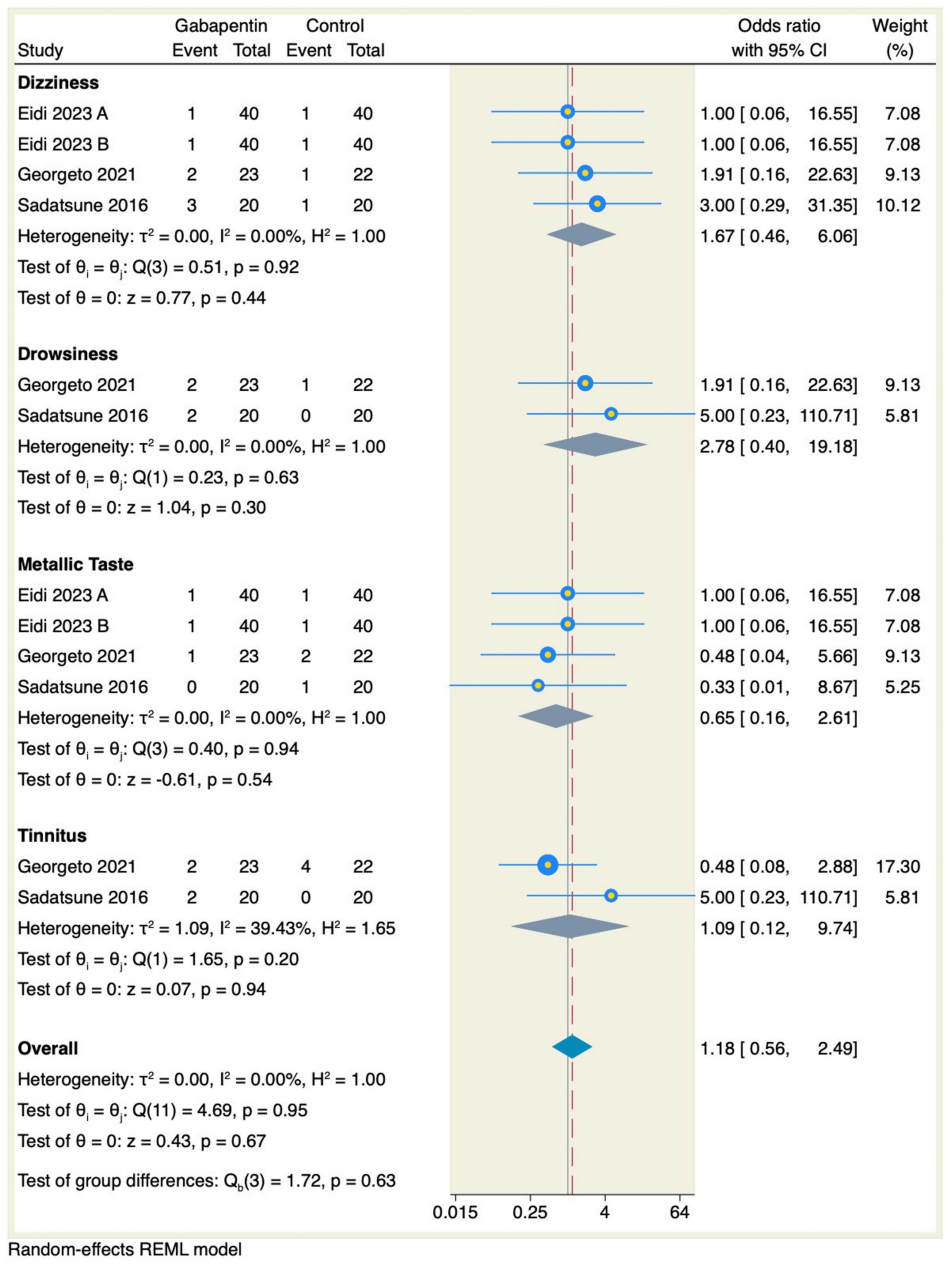
Forest plot of adverse event incidence. References: [13-15]

Regarding adverse events, there was no significant difference between gabapentin and placebo in terms of the rates of dizziness (OR: 1.67, 95% CI: 0.46 to 6.06, p = 0.44), drowsiness (OR: 2.78, 95% CI: 0.4 to 19.18, p = 0.3), metallic taste (OR: 0.65, 95% CI: 0.16 to 2.61, p = 0.54), or tinnitus (OR: 1.09, 95% CI: 0.12 to 9.74, p = 0.94), as shown in Figure 4.

Discussion

Our study is the first and the most comprehensive meta-analysis of RCTs addressing the effectiveness of gabapentin as an analgesic drug for patients with CTS undergoing release surgery including three RCTs and 205 patients. Our meta-analysis showed that gabapentin significantly reduced postoperative pain at 12 and 24 hours, with no significant change on one-hour and six-hour assessments. In addition, gabapentin showed comparable rates of adverse events compared to placebo.

Postoperative pain management is a crucial step in the surgical care of patients that not only improves the patient’s comfort and satisfaction but also reduces the risk of complications and thromboembolism and is associated with faster recovery and lower hospital costs [16]. Surgical intervention is the gold standard for severe CTS cases; however, it is associated with postoperative complications, particularly worsening pain or pain crises. Moreover, different pharmacological drugs are used preoperatively to reduce the scores of pain sensation, and gabapentin has emerged in this clinical setting due to its effect on the nociceptive process of central sensitization from pain stimulus [17].

We reported a significant decrease in postoperative pain scores at six, 12, and 24 hours compared to the placebo with no significant difference in pain scores at one hour. Our findings are in alignment with Georgeto et al. [14], and Eidi et al. [15]; however, Sadatsune et al. [13] found no significant difference between both groups in terms of postoperative pain assessment at any time point. This could be explained by the use of IVRA as their anesthetic technique, which is commonly used for the release surgery of CTS cases. However, this type of surgery can be carried out without the administration of an intra-operative anesthetic supplement as IVRA is sufficient for local anesthesia. Moreover, no sedation is required in this type of surgery as it is a fast procedure, and this can explain the mean duration of surgery, which was 33 minutes. However, due to the limitation in the duration of anesthesia, its analgesic effect is limited.

We observed a superiority of higher doses of gabapentin (600 mg versus 300 mg) in our study, and this dose-dependent effect of gabapentin to reduce postoperative pain was consistent with the literature on gabapentin’s analgesic properties [18,19]. However, the mechanism of action is not fully understood, but it can be attributed to the pharmacokinetics of the drug of which higher doses of gabapentin can lead to an enhancement of the action on calcium channels, resulting in more effective modulation [18,19].

Moreover, there were clinical differences in the inclusion criteria of Georgeto et al. [14], and Sadatsune et al. [13], which could have contributed to the difference in the findings in each study despite using a dose of 600 mg of gabapentin. The sample used by Georgeto et al. [14] was patients with bilateral idiopathic CTS, while the patients of Sadatsune et al. [13] were CTS secondary to DM in their evaluation, which could mitigate the effect of gabapentin [20]. 

Another clinical difference between the two studies was the operated hands, where patients included by Georgeto et al., presented with a severe form of CTS, unlike the operated hands of patients included by Sadatsune et al., who presented with mild to moderate CTS in 40% of the gabapentin group and 50% of the placebo group. In addition, surgical findings can be different depending on the stage of CTS pathology [21].

We demonstrated similar rates of adverse events including dizziness, drowsiness, tinnitus, and metallic taste between the gabapentin and placebo groups. These findings are in alignment with all included studies. These findings suggest that gabapentin is well-tolerated in these patients and was not associated with an increased risk of adverse events. However, it is essential to report any adverse event, especially in those patients with comorbidities such as diabetes mellitus (DM) or hypothyroidism, or those who are taking drugs that interfere with the mechanism of action of gabapentin.

The current study has some limitations for further consideration. First, the duration of pain assessment was restricted to 24 hours only, and it was the maximum point of assessment in the included studies, highlighting the need for long-term follow-up assessment beyond the acute postoperative period. Second, the analyzed data were only postoperative data, without comparison with preoperative pain scores, and further studies should highlight the assessment of preoperative pain scores before surgery. Moreover, some key clinical differences in the causes of CTS warranted further investigation; although the doses were consistent across the two studies, their values varied greatly, which could be attributed to the inclusion of their patients. Moreover, the timing of gabapentin administration varied across the studies of which two studies used gabapentin one hour postoperative and the other one used gabapentin one hour preoperative. Finally, all the included studies were single-center studies, which could limit the generalizability of the current findings, and further multicenter RCTs are warranted.

## Conclusions

This systematic review and meta-analysis, involving 205 patients, demonstrated that gabapentin effectively reduced postoperative pain at six, 12, and 24 hours, with a similar incidence of adverse events compared to placebo. Clinicians should consider using gabapentin at higher doses to enhance pain management in patients undergoing carpal tunnel release surgery. Additional RCTs are needed to confirm these findings.
